# Epigenomic Analysis Reveals the KCNK9 Potassium Channel as a Potential Therapeutic Target for Adenomyosis

**DOI:** 10.3390/ijms23115973

**Published:** 2022-05-26

**Authors:** Ling-Hui Chu, Chi-Chun Liao, Phui-Ly Liew, Chien-Wen Chen, Po-Hsuan Su, Kuo-Chang Wen, Hung-Cheng Lai, Rui-Lan Huang, Lin-Yu Chen

**Affiliations:** 1 Department of Obstetrics and Gynecology, Shuang Ho Hospital, Taipei Medical University, New Taipei City 23561, Taiwan; b89401059@ntu.edu.tw (L.-H.C.); chlitina@gmail.com (C.-C.L.); pohsuansu@gmail.com (P.-H.S.); 19345@s.tmu.edu.tw (K.-C.W.); hclai30656@gmail.com (H.-C.L.); gyntsgh@gmail.com (R.-L.H.); 2 Nuwa Fertility Center, Taipei 106467, Taiwan; 3 Department of Pathology, Shuang Ho Hospital, Taipei Medical University, New Taipei City 23561, Taiwan; lilyliew@tmu.edu.tw; 4 Department of Pathology, School of Medicine, College of Medicine, Taipei Medical University, Taipei 11031, Taiwan; 5 Dr Wang Reproductive Fertility Center, Taipei 110007, Taiwan; 017008@gmail.com; 6 Translational Epigenetics Center, Shuang Ho Hospital, Taipei Medical University, New Taipei City 23561, Taiwan; 7 Department of Obstetrics and Gynecology, School of Medicine, College of Medicine, Taipei Medical University, Taipei 11031, Taiwan; 8 Department of Obstetrics and Gynecology, School of Medicine, College of Medicine, National Defense Medical Center, Taipei 11490, Taiwan

**Keywords:** epigenomics, methylation, adenomyosis, potassium channel, KCNK9

## Abstract

Adenomyosis is linked to dysmenorrhea and infertility. The pathogenesis of adenomyosis remains unclear, and little is known of the genetic and epigenetic changes in the eutopic endometrium in adenomyosis, which may predispose patients to the invasion and migration of endometrial tissues into the myometrium. Transcriptome studies have identified genes related to various cell behaviors but no targets for therapeutic intervention. The epigenetics of the eutopic endometrium in adenomyosis have rarely been investigated. Endometrial tissue was obtained from premenopausal women with (*n* = 32) or without adenomyosis (*n* = 17) who underwent hysterectomy aged 34–57 years at a tertiary hospital. The methylome and transcriptome were assessed by using a Methylation 450 K BeadChip array and Affymetrix expression microarray. Protein expression was examined by immunohistochemistry. Differential methylation analysis revealed 53 lowly methylated genes and 176 highly methylated genes with consistent gene expression in adenomyosis, including three genes encoding potassium ion channels. High expression of KCNK9 in the eutopic and ectopic endometria in patients with adenomyosis but not in normal controls was observed. Hormone-free, antibody-based KCNK9 targeting is a potential therapeutic strategy for adenomyosis-related dysmenorrhea, menorrhagia, and infertility.

## 1. Introduction

Adenomyosis is defined as the presence of endometrial glands and stroma-like tissue within the myometrium; it is a cause of pelvic pain to many women of reproductive age. The etiology and pathology of the disease have been debated since Carl von Rokitansky first described a case of adenomyoma in 1860 [1]; moreover, its pathogenesis and role in reproduction also remain unknown. The possible mechanisms of adenomyosis include invagination of the endometrium into the myometrium, trauma to the endometrial–myometrial interface, and the stem cell potential of de novo ectopic tissue [2]. Women with adenomyosis may have an enlarged uterus and heavy or prolonged menstrual bleeding, severe menstrual cramps, or abdominal pressure and bloating. However, it is completely asymptomatic in approximately one-third of cases. The reason for such a difference in symptoms remains unknown.

For women who experience severe discomfort from adenomyosis, non-steroidal anti-inflammatory drugs and hormone therapy including progestin, oral contraceptive pills, or gonadotropin-releasing hormone (GnRH) agonists can relieve the pain and menorrhagia associated with adenomyosis to prevent the need for hysterectomy, which is the only curative treatment. Severe adenomyosis has a negative impact on a woman’s quality of life and contributes to infertility [3,4]. However, these medications may be accompanied by several side effects such as nausea, body weight gain, irregular uterine bleeding, and drug allergy. GnRH agonists are used only for short durations because they have pseudomenopausal effects such as bone loss [5,6]. Furthermore, hormone therapy does not work well in all patients and is of limited use in women who want to conceive. Therefore, new hormone-free therapies for adenomyosis-related symptoms are urgently needed.

Studies have revealed that the overexpression of genes encoding oxytocin receptors, inflammatory peptides, and neurogenic factors in the epithelia, stroma, and vessels may be correlated with dysmenorrhea in patients with adenomyosis [7,8,9,10]. The dysfunction of a group of implantation-associated factors, such as *HOXA10* and *LIF*, was identified in the eutopic endometrium in adenomyosis and led to low endometrial receptivity and subsequent infertility [11,12]. However, these findings did not lead to new therapeutics.

The pathogenesis of adenomyosis is still under debate. Sampson proposed retrograde menstruation as a mechanism in 1927, and this theory became widely accepted [1,2]. Several theories have since been proposed to clarify the pathogenic mechanisms of endometriosis, including development by metaplasia, development from Müllerian remnants, and the implantation and growth of the endometrium following retrograde menstrual reflux. However, most investigators agree that additional factors including genetic predisposition, environmental toxins, hormonal factors, and immune dysfunctions are essential for the aberrant ectopic growth of endometrial tissue [7,13]. As such, genetic or epigenetic changes may be present in the eutopic endometrium before the invasion of the myometrium in adenomyosis.

Epigenetics is the study of heritable changes in gene expression without the alteration of the DNA sequence and involves DNA methylation, histone modification, nucleosome repositioning, and gene regulation by noncoding RNAs [14,15,16]. Epigenetic changes are affected by nutrition, inflammation, lifestyle, environmental exposure, and aging [14,17,18]. DNA methylation may occur at the CpG sites of promoter regions, and it silences gene expression without changing genetic sequences; furthermore, it predisposes individuals to various diseases [14] and may be used as a biomarker for early diagnosis or patient stratification for precision treatment [19]. Several epigenetic aberrations have been discovered and suggested to play a role in the development of endometriosis. Altered methylation of HOX clusters was identified in the ectopic and eutopic endometrial tissues and organoids of patients with endometriosis compared with those with a normal endometrium [20]. Reduced expression of *HDAC3*, which serves as a histone deacetylase, is associated with endometriosis and decreased endometrial receptivity [21]. However, the epigenetic studies on adenomyosis remain few.

In the present study, we hypothesized that epigenetic changes occur in the eutopic endometrium in adenomyosis and therefore may lead to novel therapeutic strategies.

## 2. Results

### 2.1. Differential Methylomic and Transcriptomic Profiles of Eutopic Endometrial Tissues in Adenomyosis and Control Groups

**Figure 1:**
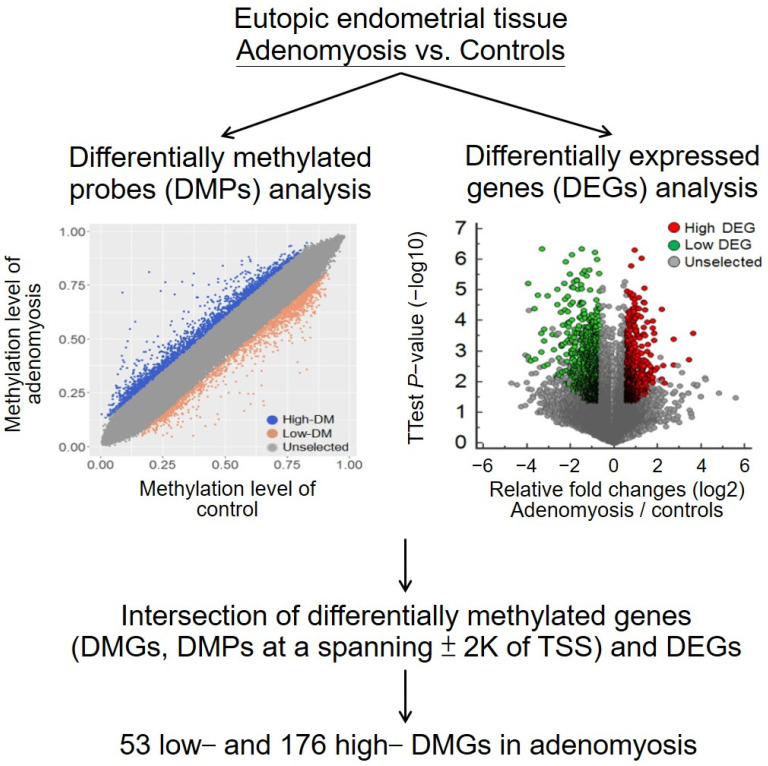
The flowchart of the analytical strategy. Differential methylomic and transcriptomic profiles of eutopic endometrial tissues between patients with adenomyosis and controls. DMPs, differentially methylated probes; DEGs, differentially expressed genes; DMGs, differentially methylated genes; TSS, transcriptional start site.

To identify differentially methylated genes (DMGs) in adenomyosis, we generated and compared the methylomic profiles of the eutopic endometrium in patients with adenomyosis (*n* = 5) and controls (*n* = 2) (Table S1). The study flowchart is presented in Figure 1. The methylomic profiles were constructed using a methylation 450K BeadChip. This BeadChip probe was used to detect the methylation level at a CpG site. According to the CpG loci related to the closest coding gene, major changes in the CpG site distribution were located at the promoter (including the 5′ untranslated region (UTR)), gene body, and 3′ UTR. The distributions of lowly methylated CpG sites in the adenomyosis group were 25%, 37%, and 29% at the promoter, gene body, and intergenic regions, respectively, whereas for highly methylated CpG sites, they were 30%, 35%, and 30%. The percentage at promoter regions was higher for highly methylated CpG sites than for lowly methylated CpG sites.

**Figure 2:**
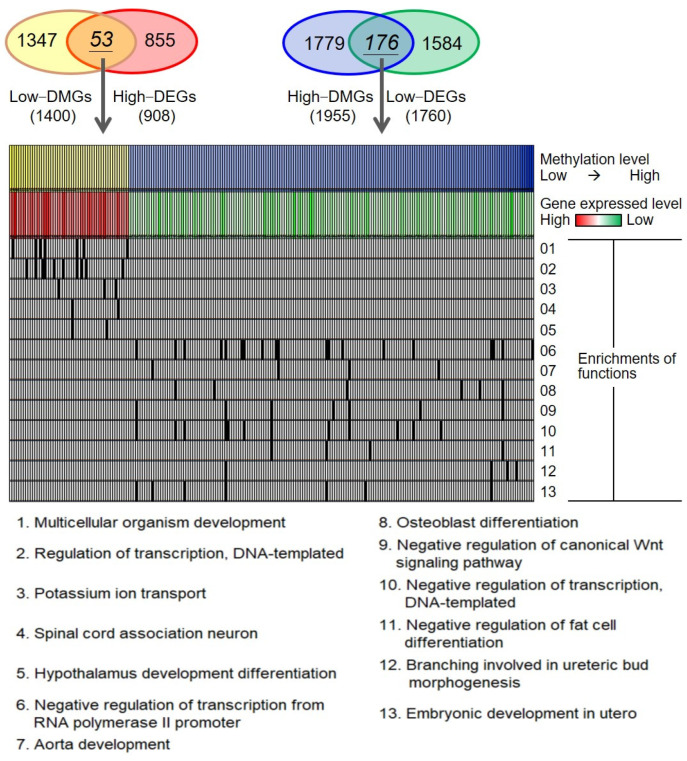
Functional enrichment analysis and heatmap for differentially methylated and expressed profiles. Venn diagrams show selected genes with a negative correlation between methylation and gene expression in patients with adenomyosis and controls. Items 01–05 list the enriched biological functions of 53 lowly DMGs and highly DEGs as multicellular organism development, regulation of transcription, and potassium ion transport. Items 06–13 list the enriched biological functions of 176 highly DMGs and lowly DEGs as negative regulation of transcription from RNA polymerase II promoter, aorta development, osteoblast differentiation, and negative regulation of the canonical Wnt signaling pathway. DEGs, differentially expressed genes; DMGs, differentially methylated genes.

Not all the DMGs correlated with gene expression changes. Therefore, we identified 1955 highly differentially expressed genes (DEGs) and 1400 lowly DEGs in adenomyosis from a public dataset. From the intersection of the DMGs and DEGs, we selected 53 lowly DMGs with high expression and 176 highly DMGs with low expression in adenomyosis, as shown in Figure 1 and the upper panel of Figure 2.

### 2.2. Biological Functions of Adenomyosis-Associated Epigenetic Changes

In order to understand DNA-methylation-mediated deregulations in adenomyosis, we annotated the DMGs and discovered the enriched functions (lower panel of Figure 2). The enriched biological functions of 53 lowly DMGs and highly DEGs were multicellular organism development, regulation of transcription, and potassium ion transport, which may be related to the pathogenesis of adenomyosis. A total of 176 highly DMGs/lowly DEGs were predominantly enriched in the negative regulation of transcription from RNA polymerase II promoter, aorta development, osteoblast differentiation, and negative regulation of canonical Wnt signaling pathway, which may play a role in diagnosis.

### 2.3. Verification of Hypomethylated Genes

**Figure 3:**
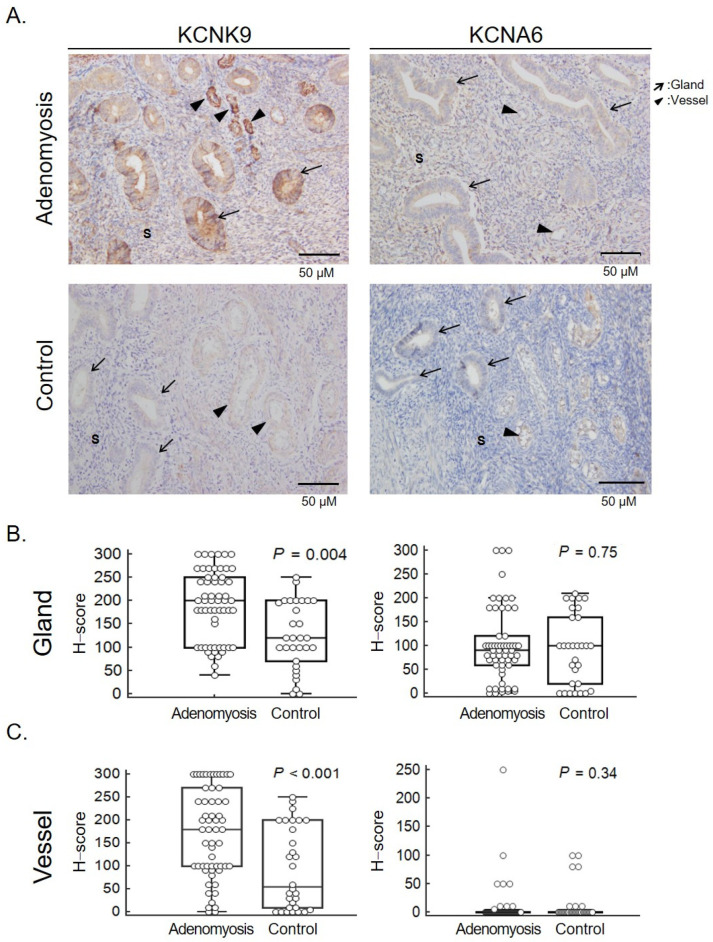
KCNK9 and KCNA6 expression in the eutopic endometrium of patients with adenomyosis and controls. (**A**) Representative immunohistochemical (IHC) staining of KCNK9 and KCNA6 proteins. Endometrial glands and vessels are indicated by arrows and triangles, respectively. S, stroma. Comparison of KCNK9 and KCNA6 expression in endometrial glands (**B**) and vessels (**C**). The expression was scored as follows: intensity x area of positively stained cells (%). Significant high expression of KCNK9 in the endometrial glands (*p* = 0.004) and endometrial vessels (*p* < 0.001) in adenomyosis.

**Figure 4:**
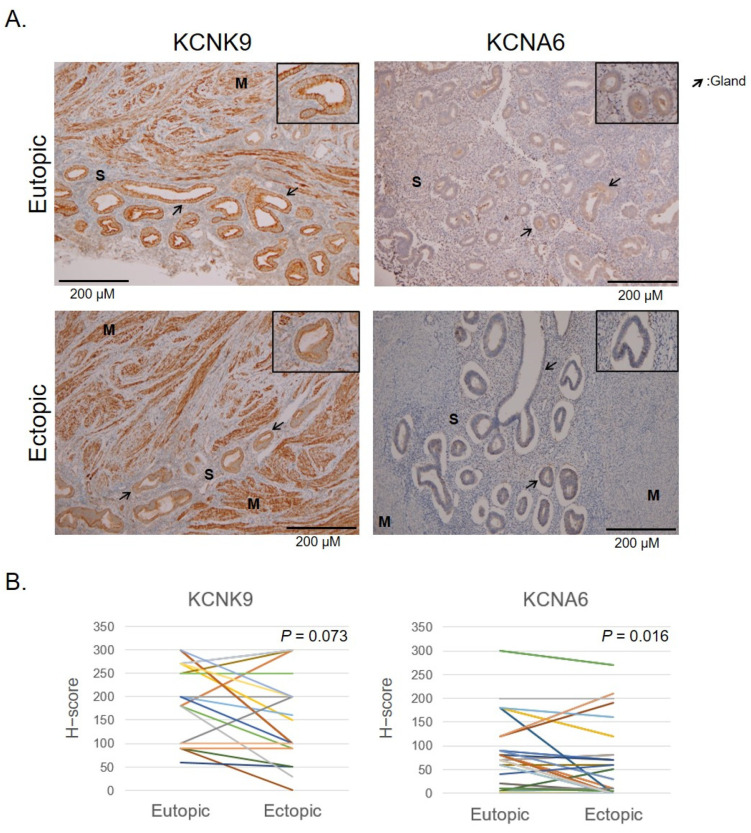
KCNK9 and KCNA6 expression in the ectopic endometrium in adenomyosis. (**A**) Representative IHC staining of KCNK9 and KCNA6 proteins. Endometrial glands (indicated by arrows). S, stroma; M, muscle. (**B**) Comparison of expression of KCNK9 and KCNA6 in ectopic and eutopic endometria. A significant difference was observed in the deceased KCNA6 in the ectopic endometrium (*p* = 0.016, paired non-parametric Wilcoxon signed-rank test).

In order to develop potential therapeutic targets, we selected the potassium ion channel genes KCNMB3, KCNA6, and KCNK9 for further verification, with PAX8 as the positive control and WNT5A as the negative control. KCNMB3 was not detected in IHC. KCNA6 and KCNK9 were associated with potassium ion transport, which was associated with pain and cell migration [22,23]. We verified the protein levels of candidate genes in the eutopic and ectopic endometrium from patients with adenomyosis and controls by using IHC staining. Comparisons of the staining intensity and proportion were made among the functional and basal layers of adenomyotic nodules and the eutopic endometrium in the adenomyosis and control groups (Figure 3A). The calculated H-score is illustrated in Figure 3B,C. KCNK9 expression was increased in the adenomyosis group compared with the control group. A significant difference was discovered among the endometrial glands (*p* = 0.004) and endometrial vessels (*p* < 0.001) in the adenomyosis group compared with the control group. The KCNK9 expression in the endometrial stroma was not significantly different among those with and without adenomyosis (*p* = 0.174). The KCNK9 expression was not significantly different in the gland cells between the ectopic and eutopic endometria in adenomyosis (Figure 4). The KCNA6 expression in the ectopic endometrium was as weak as it was in the eutopic endometrium. However, in paired nonparametric Wilcoxon signed-rank tests, we found a significant decrease in KCNA6 expression in the ectopic endometrium (*p* = 0.016). The KCNK9 protein was DNA-methylated. Hypomethylated KCNK9 exhibited a higher protein level in the gland cells and vessel epithelia in patients with adenomyosis.

### 2.4. Verification of Hypermethylated Genes

**Figure 5:**
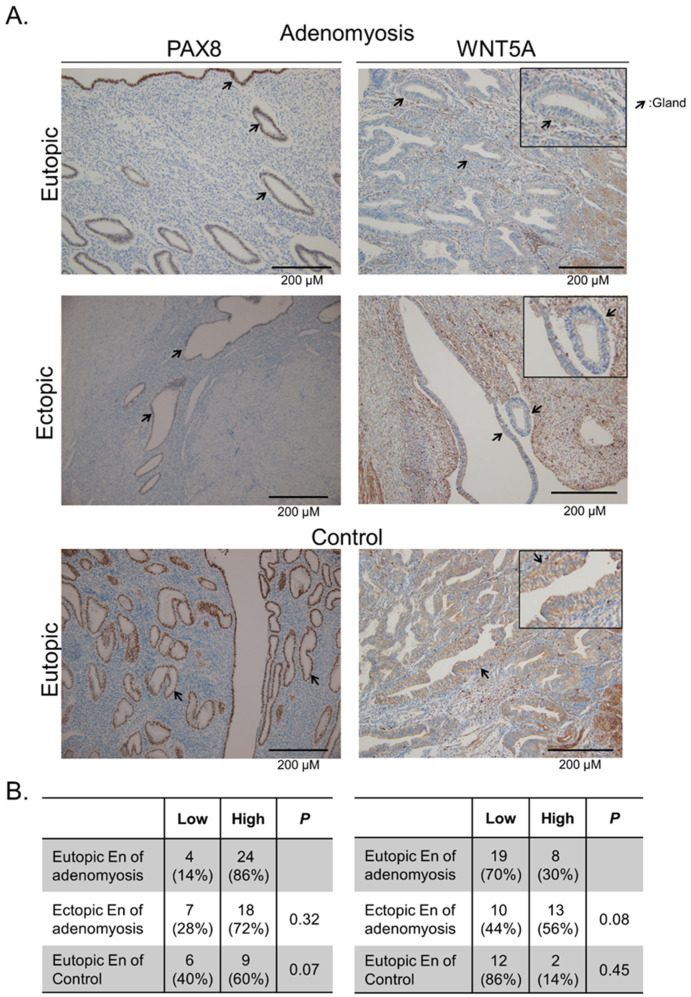
PAX8 and WNT5A in the eutopic and ectopic endometrium of patients with adenomyosis and controls. (**A**) Representative IHC staining of the PAX8 and WNT5A proteins. (**B**) Proportion of PAX8 and WNT5A intensity in the endometrial gland cells is shown. In the functional endometrium, high expression of PAX8 and WNT5A was shown. A lower percentage of WNT5A with low expression was detected in adenomyosis.

Two hypermethylated genes were selected for the verification of protein expression in IHC in the same manner as that for hypomethylated genes (Figure 5). PAX8 and WNT5A are associated with the development of the functional endometrium [24,25,26]. In the adenomyosis and control groups, most of the samples exhibited high PAX8 expression in the functional layer of the endometrium (the proportions of high expression: adenomyosis, 86%; control, 60%; *p* = 0.07). WNT5A exhibited weak-to-moderate expression in both the gland and stromal cells in the adenomyosis and control groups (the proportions of high expression: adenomyosis, 30%; control, 14%; *p* = 0.45). Unlike for the low expression of gene products from hypermethylated PAX8 and WNT5A in adenomyosis, the IHC staining results revealed inconsistent trends in higher protein expression. The results imply that other factors apart from DNA methylation play a vital role in PAX8 and WNT5A production.

## 3. Discussion

Our study revealed that the methylomic profiles of the eutopic endometrium were different between the adenomyosis and control groups. KCNK9, a subgroup of the potassium ion channel protein, was highly expressed in the endometrium of patients with adenomyosis through DNA demethylation. This study is the first genome-wide methylation investigation of adenomyosis and revealed a novel ion channel, KCNK9, that may lead to new hormone-independent therapeutic strategies.

Recent studies have shown that adenomyosis is a type of metastatic disease similar to malignant tumors and has some aspects of malignancy such as angiogenesis, invasion, planting, and recurrence [27]. Our functional enrichment analysis also identified genes involved in negative regulation of the canonical Wnt signaling pathway (Table S2), which plays a crucial role in tumor maintenance, metastasis, and immune evasion [28]. For example, the tumor-suppression gene programmed cell death 4 was downregulated in adenomyosis [29], and kisspeptin 1 is a metastasis-suppressor gene that exhibited high expression and may serve as a biomarker in adenomyosis [30]. These results imply some malignant behaviors in adenomyosis.

Transient changes in ionic distribution alter the membrane potential, and this forms the basis for various biological processes. Potassium channels are the most widely distributed type of ion channel and are found in virtually all living organisms. They are also key regulators of neurons and muscle cells and have been reported to be involved in cell migration and pain. There are four major classes of potassium channel: calcium-activated potassium channels, inward-rectifier potassium channels, two-pore domain potassium (K2P) channels, and voltage-gated potassium channels (VGKCs). KCNK9 is a member of the K2P channel family, and KCNA6 is a member of the VGKC family [31]. Genetic evidence implicates KCNK9 in cancer. However, it has not been considered relevant to adenomyosis. Our results demonstrated that KCNK9 was highly expressed in both the eutopic and ectopic endometria in adenomyosis. The hypomethylation of these channels in the eutopic endometrium in adenomyosis may be related to endometrial cell migration to the myometrium with invasion. High expression in adenomyotic nodules may be related to dysmenorrhea through the loss of normal rhythmic contraction and disturbed uterine receptivity due to hyperplastic muscular tissue and dysfunctional uterine hyperperistalsis. Sun et al. developed monoclonal antibodies against the special KCNK9 extracellular domain by leveraging the domain’s poor sensitivity to common ion channel blockage and proved that they could inhibit lung and breast cancer cell growth in vivo [32]. This provides insight into the physiological and pathological significance of adenomyosis. Antibody-based KCNK9 targeting may be a promising therapeutic strategy in adenomyosis.

There are limitations to the present study. First, all the patients with adenomyosis had dysmenorrhea and menorrhagia; the association between KCNK9 and adenomyosis-related symptoms has not yet been established. Further investigation of KCNK9 in the endometria of women with and without symptoms may clarify the correlation between this ion channel and symptoms. Second, our cross-sectional study was limited in its ability to indicate a causal relationship between KCNK9 expression in the eutopic endometrium and the development of adenomyosis. Additionally, we collected endometrial samples in either the secretory or proliferative phase of the endometrium, which may have been a confounding factor in the IHC results. We performed a sub-analysis according to endometrial phases, and still found significantly higher expression of the KCNK9 protein on the eutopic endometrium in adenomyosis in both the proliferative and secretory subgroups.

## 4. Materials and Methods

### 4.1. Sample Collection

Endometrial tissues were obtained from women with (*n* = 32) or without (*n* = 17) adenomyosis who underwent hysterectomy (Table S1). All the participants were premenopausal and aged 34–57 years; the patient demographics are listed in Table S1. All the patients with adenomyosis had symptoms of both dysmenorrhea and menorrhagia. The diagnosis of adenomyosis was confirmed through histopathological examination after the operation. The eutopic endometrial specimens were free of endometrial pathology, such as polyps or hyperplasia. All the patients with adenomyosis had the symptoms dysmenorrhea, pelvic pain, and/or menorrhagia. The exclusion criteria were hormone treatment at least 3 months before the surgery, pregnancy, and reproductive tract cancer. All the participants provided written informed consent for joining the Taipei Medical University Joint Biobank (TMU-JBB). All the residual specimens and tissue sections were obtained in accordance with the Declaration of Helsinki, and all the protocols were approved by the institutional review board of the TMU-JIRB.

### 4.2. DNA Extraction and Bisulfite Conversion

Genomic DNA was extracted from endometrial tissues using the QIAamp DNA Mini Kit (QIAGEN, Hilden, Germany). Bisulfite modification (800 ng of DNA) was performed using the EZ DNA Methylation Kit (D5008; Zymo Research, Irvine, CA, USA) in accordance with the manufacturer’s recommendations, dissolving the sample in 70 μL of nuclease-free water. For the construction of the methylomic profiles, we purified the bisulfite-converted DNA to obtain more than 0.5 μg of DNA by using a QIAamp Clean Kit (QIAGEN, Hilden, Germany). DNA samples with absorbance ratios of >1.8 at 260/280 and 260/230 met the quality standards.

### 4.3. Methylomic and Transcriptomic Data Analysis

We obtained the methylomic profiles of the endometrial tissues in the adenomyosis (*n* = 5) and control (*n* = 2) groups on a Methylation 450K BeadChip array (Illumina, Inc., San Diego, CA, USA) (Table S1). An equal amount of endometrial DNA from each individual with adenomyosis and each control was used to create two pooled DNA samples. For the differentially methylated genes (DMGs), a between-group difference of ≥10% in the mean β values for the methylation status for each probe was allowed. Each probe detected the methylation level of a CpG site. We focused on the CpG sites located closest (≤2000 bp) to the transcriptional start sites (TSSs) of the coding genes, including ≤2 single nucleotide polymorphisms of the probes and excluding CpG sites on the sex chromosome. If a gene had more than one differentially methylated probe, we selected the maximum differential level.

We integrated the transcriptomic profiles of the eutopic endometrium from women with adenomyosis (*n* = 3) and without (*n* = 5); the samples were flash-frozen in liquid nitrogen, and the RNA was extracted and transcribed into cDNA and amplified, which was analyzed with the Human Gene 1.0 ST Array (Affymetrix, Santa Clara, CA, USA); records were retrieved from the Gene Expression Omnibus, and assessed with respect to GSE78851 [33]. The significant differential gene expression allowed for ≥1.5-fold changes in the quantified expression level, with a *p* value of ≤0.05 as per the two-tailed Student’s t-test and excluding the sites on the sex chromosome. We focused on highly DMGs with low gene expression and lowly DMGs with high gene expression.

### 4.4. Functional Enrichment Analysis

To study selected genes at a functional level, functional enrichment analysis was performed using the online biological tool Database for annotation, visualization, and integrated discovery (DAVID version 6.8; https://david.ncifcrf.gov/; accessed on 22 November 2019) [34] (Table S2). DAVID has been extensively used to identify biological processes of gene ontology involving a given list of genes. In the present study, enriched terms with more than two genes and a modified Fisher’s exact *p*-value of <0.05 were considered statistically significant.

### 4.5. Immunohistochemistry Staining

To evaluate the protein expression in the endometrium in the patients with adenomyosis and controls, we performed immunohistochemistry (IHC) staining in paraffin-embedded sections. The samples were evaluated for potassium ion proteins by using rabbit polyclonal antibodies to KCNA6 (1:200 dilution, PA5-53163, Thermo Fisher Scientific, Waltham, MA, USA), KCNK9 (1:100 dilution, PA5-41044, Thermo Fisher Scientific, Waltham, MA, USA), PAX8 (MRQ-50, MilliporeSigma, Darmstadt, Germany), and WNT5A (1:1200 dilution, LS-B3859, LSBio, Seattle, WA, USA). The expressed levels of KCNA6 and KCNK9 were scored by two pathologists, and the expression score was calculated as the product of the intensity and area of positively stained cells. The intensity was scored as 0 (no staining), 1 (weak), 2 (moderate), or 3 (strong). The area of positively stained cells was scored from 0% (<10% of counted cells) to 100% (all positive). We calculated the H-score by multiplying the intensity by the total percentage of the stained area.

### 4.6. Statistical Analysis

The Kruskal–Wallis test was used to identify differences in the methylation level between two categories, and the post hoc rank-sum test was then performed. Significant differences were assessed using a two-tailed *p*-value of ≤0.05. All the analyses were performed and plots obtained using MedCalc (version 17, Acacialaan 22, 8400 Ostend, Belgium).

## 5. Conclusions

This is the first study to report the association of KCNK9 with adenomyosis. KCNK9 was demethylated and highly expressed in the eutopic and ectopic endometria in adenomyosis, which indicates a new field of adenomyosis research. A nonhormonal KCNK9-targeting therapy may hold therapeutic promise. Clinical trials on KCNK9-targeting therapy for symptomatic adenomyosis are warranted.

## Data Availability

The data have been deposited in the GEO database under the accession code GSE78851.

## Supplementary Materials

The following supporting information can be downloaded at: https://www.mdpi.com/article/10.3390/ijms23115973/s1.

## Abbreviations

GnRH	gonadotropin-releasing hormone
UTR	untranslated region
DEGs	differentially expressed genes
K2P	two-pore domain potassium
VGKCs	voltage-gated potassium channels
TSS	transcriptional start site
IHC	immunohistochemistry
FFPE	formalin-fixed paraffin-embedded
