# Supplementary material for: Epigenomic Analysis Reveals the KCNK9 Potassium Channel as a Potential Therapeutic Target for Adenomyosis
Source: Int J Mol Sci. 2022 May 26;23(11):5973. doi: 10.3390/ijms23115973 (PMC9180761; doi:10.3390/ijms23115973)
Supplement: Supplementary file 1 [file ijms-23-05973-s001.zip › ijms-1734501-supplementary.pdf]

Table S1. Demographic of endometrium for immunohistochemistry analysis

Case ID	Diagnosis	Endometrium status	Age	Specimen type	Mean ± SD	P value*
For discovery						
MAr-En-001	Adenomyosis	proliferative	40	Frozen	45.7 ± 3.5	0.91
MAr-En-002	Adenomyosis	weakly proliferative	42	Frozen		
MAr-En-003	Adenomyosis	proliferative	48	Frozen		
MAr-En-004	Adenomyosis	weakly proliferative	54	Frozen		
MAr-En-005	Adenomyosis	proliferative	45	Frozen		
MAr-En-006	Fibroids	proliferative	48	Frozen	45.3 ± 3.5	
MAr-En-007	Fibroids	proliferative	43	Frozen		
For validation						
IHC-En-002	Adenomyosis	early-secretory	39	FFPE	46.7 ± 8.4	0.99
IHC-En-003	Adenomyosis	mid-secretory	46	FFPE		
IHC-En-004	Adenomyosis	mid-secretory	48	FFPE		
IHC-En-005	Adenomyosis	mid-secretory	35	FFPE		
IHC-En-006	Adenomyosis	mid-secretory	50	FFPE		
IHC-En-007	Adenomyosis	mid-secretory	52	FFPE		
IHC-En-008	Adenomyosis	mid-secretory	45	FFPE		
IHC-En-009	Adenomyosis	late-secretory	49	FFPE		
IHC-En-010	Adenomyosis	late-secretory	43	FFPE		
IHC-En-011	Adenomyosis	late-secretory	34	FFPE		
IHC-En-012	Adenomyosis	late-secretory	43	FFPE		
IHC-En-013	Adenomyosis	proliferative	49	FFPE		
IHC-En-014	Adenomyosis	proliferative	53	FFPE		
IHC-En-015	Adenomyosis	proliferative	45	FFPE		
IHC-En-016	Adenomyosis	proliferative	41	FFPE		
IHC-En-017	Adenomyosis	proliferative	44	FFPE		
IHC-En-018	Adenomyosis	proliferative	44	FFPE		
IHC-En-019	Adenomyosis	proliferative	55	FFPE		
IHC-En-021	Adenomyosis	proliferative	34	FFPE		
IHC-En-022	Adenomyosis	proliferative	47	FFPE		
IHC-En-023	Adenomyosis	proliferative	43	FFPE		
IHC-En-024	Adenomyosis	proliferative	39	FFPE		
IHC-En-025	Adenomyosis	proliferative	54	FFPE		
IHC-En-026	Adenomyosis	weakly proliferative	39	FFPE		
IHC-En-027	Adenomyosis	weakly proliferative	57	FFPE		
IHC-En-028	Adenomyosis	weakly proliferative	40	FFPE		
IHC-En-029	Adenomyosis	weakly proliferative	53	FFPE		
IHC-En-030	Fibroids	mid-secretory	44	FFPE	47 ± 4.2	
IHC-En-031	Fibroids	mid-secretory	44	FFPE		
IHC-En-032	Fibroids	late-secretory	48	FFPE		
IHC-En-033	Fibroids	late-secretory	44	FFPE		
IHC-En-034	Fibroids	proliferative	44	FFPE		
IHC-En-035	Fibroids	proliferative	45	FFPE		
IHC-En-036	Fibroids	proliferative	47	FFPE		
IHC-En-037	Fibroids	proliferative	52	FFPE		
IHC-En-038	Fibroids	proliferative	49	FFPE		
IHC-En-039	Fibroids	proliferative	42	FFPE		
IHC-En-040	Fibroids	proliferative	41	FFPE		
IHC-En-041	Fibroids	weakly proliferative	56	FFPE		
IHC-En-042	Fibroids	weakly proliferative	52	FFPE		
IHC-En-043	Fibroids	weakly proliferative	48	FFPE		
IHC-En-044	Fibroids	weakly proliferative	49	FFPE		

*Two-tailed Welch-test. FFPE, formalin-fixed, paraffin-embedded.

Table S2. Functional enrichments of selected genes

GO	Gene Name	P-value
**Low methylation and high gene expression in adenomyosis**		
1. Multicellular organism development	*ZAR1, HOXC12, LMO2, ZNF541, PRM1, LIMD1, NKX2-4*	3.9E-03
2. Regulation of transcription, DNA-templated	*HOXC12, BRF1, SOX1, HINT1, NKX6-2, SP5, LIMD1, NKX2-4, FZD7, ZNF23*	1.0E-02
3. Potassium ion transport	*KCNMB3, KCNK9, KCNA6*	2.4E-02
4. Spinal cord association neuron	*TAL1, GSX1*	3.7E-02
5. Hypothalamus development differentiation	*RAX, GSX1*	4.3E-02
**High methylation and low gene expression in adenomyosis**		
1. Negative regulation of transcription from RNA polymerase II promoter	*BACH1, AEBP1, ZNF593, HIST1H1D, KLF11, FHL2, DUSP22, SMAD3, WHSC1, REST, WWTR1, GLI3, CHD8, SIN3A, ATN1, HEYL, GATAD2B, PTCH1, PRDM1*	1.2E-04
2. Aorta development	*LTBP1, PRICKLE1, PRDM1, MYH10*	5.7E-04
3. Osteoblast differentiation	*MRC2, FHL2, COL6A1, WWTR1, GPNMB, IGFBP3*	2.8E-03
4. Negative regulation of canonical Wnt signaling pathway	*WNT5A, CHD8, PPP2R3A, PRICKLE1, BICC1, WWTR1, GLI3*	4.1E-03
5. Negative regulation of transcription, DNA-templated	*WNT5A, CHD8, SIN3A, PRICKLE1, KLF11, HEYL, POU2F1, KCTD1, FHL2, LRRFIP1, REST, GLI3*	6.7E-03
6. Negative regulation of fat cell differentiation	*WNT5A, SMAD3, ZADH2, WWTR1*	6.7E-03
7. branching involved in ureteric bud morphogenesis	*PAX8, PTCH1, GLI3, HOXD11*	6.7E-03
8. in utero embryonic development	*CHD8, SIN3A, SYF2, SMAD3, PTCH1, GLI3, MYH10*	7.8E-03
